# Vasopressin V1a receptors mediate the hypertensive effects of [Pyr^1^]apelin‐13 in the rat rostral ventrolateral medulla

**DOI:** 10.1113/JP274178

**Published:** 2017-04-21

**Authors:** Philip R. Griffiths, Stephen J. Lolait, Louise E. Harris, Julian F. R. Paton, Anne‐Marie O'Carroll

**Affiliations:** ^1^ School of Clinical SciencesUniversity of BristolBristolUK; ^2^ School of Physiology, Pharmacology and Neuroscience, Biomedical SciencesUniversity of BristolBristolUK

**Keywords:** apelin, APJ, blood pressure, rostral ventrolateral medulla, sympathetic nervous system, vasopressin

## Abstract

**Key points:**

- Dysfunctions in CNS regulation of arterial blood pressure lead to an increase in sympathetic nerve activity that participates in the pathogenesis of hypertension.
- The apelin‐apelin receptor system affects arterial blood pressure homeostasis; however, the central mechanisms underlying apelin‐mediated changes in sympathetic nerve activity and blood pressure have not been clarified.
- We explored the mechanisms involved in the regulation of [Pyr^1^]apelin‐13‐mediated cardiovascular control within the rostral ventrolateral medulla (RVLM) using selective receptor antagonists.
- We show that [Pyr^1^]apelin‐13 acts as a modulating neurotransmitter in the normotensive RVLM to affect vascular tone through interaction with the vasopressin V1a receptor but that [Pyr^1^]apelin‐13‐induced sympathoexcitation is independent of angiotensin II receptor type 1, oxytocin, ionotropic glutamate and GABA_A_ receptors.
- Our data confirm a role for the apelin peptide system in cardiovascular regulation at the level of the RVLM and highlight that this system is a possible potential therapeutic target for the treatment of hypertension.

**Abstract:**

Apelin is a ubiquitous peptide that can elevate arterial blood pressure (ABP) yet understanding of the mechanisms involved remain incomplete. Bilateral microinjection of [Pyr^1^]apelin‐13 into the rostral ventrolateral medulla (RVLM), a major source of sympathoexcitatory neurones, increases ABP and sympathetic nerve activity. We aimed to investigate the potential involvement of neurotransmitter systems through which the apelin pressor response may occur within the RVLM. Adult male Wistar rats were anaesthetized and ABP was monitored via a femoral arterial catheter. Bilateral RVLM microinjection of [Pyr^1^]apelin‐13 significantly increased ABP (9 ± 1 mmHg) compared to saline (−1 ± 2mmHg; *P* < 0.001), which was blocked by pretreatment with the apelin receptor antagonist, F13A (0 ± 1 mmHg; *P* < 0.01). The rise in ABP was associated with an increase in the low frequency spectra of systolic BP (13.9 ± 4.3% total power; *P* < 0.001), indicative of sympathetic vasomotor activation. The [Pyr^1^]apelin‐13‐mediated pressor response and the increased low frequency spectra of systolic BP response were fully maintained despite RVLM pretreatment with the angiotensin II type 1 receptor antagonist losartan, the oxytocin receptor antagonist desGly‐NH_2_, d(CH_2_)_5_[D‐Tyr^2^,Thr^4^]OVT, the ionotropic glutamate receptor antagonist kynurenate or the GABA_A_ antagonist bicuculline (*P *> 0.05). By contrast, the [Pyr^1^]apelin‐13 induced pressor and sympathoexcitatory effects were abolished by pretreatment of the RVLM with the vasopressin V1a receptor antagonist, SR 49059 (−1 ± 1 mmHg; 1.1 ± 1.1% total power, respectively; *P* < 0.001). These findings suggest that the pressor action of [Pyr^1^]apelin‐13 in the RVLM of normotensive rats is not mediated via angiotensin II type 1 receptor, oxytocin, ionotropic glutamate or GABA_A_ receptors but instead involves a close relationship with the neuropeptide modulator vasopressin.

## Key points

- Dysfunctions in CNS regulation of arterial blood pressure lead to an increase in sympathetic nerve activity that participates in the pathogenesis of hypertension.
- The apelin‐apelin receptor system affects arterial blood pressure homeostasis; however, the central mechanisms underlying apelin‐mediated changes in sympathetic nerve activity and blood pressure have not been clarified.
- We explored the mechanisms involved in the regulation of [Pyr^1^]apelin‐13‐mediated cardiovascular control within the rostral ventrolateral medulla (RVLM) using selective receptor antagonists.
- We show that [Pyr^1^]apelin‐13 acts as a modulating neurotransmitter in the normotensive RVLM to affect vascular tone through interaction with the vasopressin V1a receptor but that [Pyr^1^]apelin‐13‐induced sympathoexcitation is independent of angiotensin II receptor type 1, oxytocin, ionotropic glutamate and GABA_A_ receptors.
- Our data confirm a role for the apelin peptide system in cardiovascular regulation at the level of the RVLM and highlight that this system is a possible potential therapeutic target for the treatment of hypertension.

**Abbreviations**
Ang II: angiotensin II
APJ: apelin receptor
BP: blood pressure
HR: heart rate
HRV: heart rate variability
MABP: mean arterial blood pressure
NTS: nucleus tractus solitarius
OT: oxytocin
dOVT: desGly‐NH_2_, d(CH_2_)_5_[D‐Tyr^2^,Thr^4^]OVT
PVN: parvocellular paraventricular nucleus
RVLM: rostral ventrolateral medulla
SBPV: systolic blood pressure variability
SHR: spontaneously hypertensive rats
SNA: sympathetic nerve activity
SON: supraoptic nucleus
VP: vasopressin
WKY: Wistar Kyoto

## Introduction

Dysfunctions in CNS regulation of blood pressure (BP) lead to an increase in sympathetic nerve activity (SNA) that participates in the pathogenesis of hypertension (Esler *et al*. 2001). Chronic sympathetic hyperactivity is present in a significant percentage of patients with essential hypertension, and is responsible for more than 90% of all hypertensive disorders (Carretero & Oparil, 2000). The causative mechanisms of elevated SNA remain unclear, although they probably involve both neural and humoral mechanisms. Studies have implicated the apelin‐apelin receptor (APJ) system in the increase of SNA and in the development of hypertension (Wu *et al*. 2014), although the underlying mechanism of action is undetermined.

Apelin, a 36 amino acid peptide, is synthesized as part of a 77‐amino acid preproapelin precursor originally isolated from bovine stomach extracts (Tatemoto *et al*, 1998) and mediates its effects via the G protein‐coupled apelin receptor, APJ (O'Dowd *et al*. 1993; Kawamata *et al*. 2001; O'Carroll *et al*. 2013). In addition to apelin‐36, other *in vivo* isoforms of the apelin peptide, such as apelin‐12, apelin‐13 [including pyroglutamyl apelin‐13 ([Pyr^1^]apelin‐13), the predominant isoform within the cardiovascular system (Maguire *et al*. 2009)] and apelin‐17 also bind to and activate APJ (Kawamata *et al*. 2001). Apelin‐36 and ‐13 may be the most abundant and biologically active fragments (Kawamata *et al*. 2001). APJ mRNA expression and apelin immunoreactivity are present in central neural circuits and lower brainstem structures that are involved in the modulation of BP, including the medial parvocellular paraventricular nucleus (PVN), the rostral ventrolateral medulla (RVLM) and the nucleus tractus solitarius (NTS) (O'Carroll *et al*. 2013), and are known regulators of cardiovascular (Reaux *et al*. 2001) and fluid homeostasis (O'Carroll & Lolait 2003).

Apelin expression is enhanced in the RVLM of spontaneously hypertensive rats (SHR), a genetic model of hypertension, compared to normotensive Wistar Kyoto (WKY) rats (Zhang *et al*. 2009b) and microinjection of an apelin‐neutralizing antibody into the RVLM of SHR significantly lowers BP (Zhang *et al*. 2009b) in SHR, suggesting that the apelinergic system may be up‐regulated in hypertensive disease. Moreover targeted injection of [Pyr^1^]apelin‐13 into the RVLM or parvocellular PVN, or by i.c.v. injection, demonstrates increased mean arterial blood pressure (MABP) and sympathetic nervous system activity in the rat (Zhang *et al*. 2009b, 2014; Kagiyama *et al*. 2005). These studies indicate that apelin is sympathoexcitatory in the RVLM and that this as yet unclarified mechanism is endogenously active in the hypertensive model. A number of neuronal inputs containing excitatory and inhibitory neurotransmitters converge on RVLM sympathoexcitatory neurons from multiple areas of the brain and spinal cord to control BP by regulation of SNA; for example, endogenous angiotensin II (AngII) (Kumagai *et al*. 2012) or vasopressin (VP) released from PVN neurons (Kc *et al*. 2010) potentiate RVLM neurons, whereas GABAergic inhibitory input from caudal ventrolateral medulla neurons suppresses RVLM neuronal activity (Kumagai *et al*. 2007), and these opposing influences regulate its neuronal excitability. APJ has closest identity to the Ang II receptor type AT1a (∼54% in transmembrane regions) (O'Dowd *et al*. 1993) and the receptors share a similar anatomical distribution (Medhurst *et al*. 2003), although APJ does not bind Ang II and the effects of apelin are independent of AT1a (Wu *et al*. 2014; O'Carroll *et al*. 2013). Nevertheless, a direct interaction between these receptor systems has been described, with apelin and APJ acting as counter‐regulators of the renin‐angiotensin‐aldosterone system (Siddiquee *et al*. 2013). Additionally, APJ mRNA expression and apelin immunoreactivity are co‐localized with VP (Reaux *et al*. 2001; O'Carroll & Lolait, 2003) and oxytocin (OT) (Brailoiu *et al*. 2002) in magnocellular cells of both the PVN and supraoptic nucleus (SON). We have shown, in previous studies, that [Pyr^1^]apelin‐13 increases the firing rates of VP neurons in the SON (Tobin *et al*. 2008) and also that the *in vivo* effects of apelin on both hypothalamic‐neurohypophysial system (Roberts *et al*., 2010) and hypothalamic‐pituitary‐adrenal axis (Newson *et al*. 2009) neuroendocrine function are mediated through VP‐dependent mechanisms. Furthermore, apelin significantly stimulates VP release from rat hypothalamic explants (Taheri *et al*. 2002). Therefore, possible synaptic mechanisms connecting apelin/APJ with these peptidergic systems may exist.

Considering the localization of apelin and APJ within the cardiovascular neural centres, we hypothesized that apelin may act as a modulatory neurotransmitter locally and/or via volume transmission to affect neurotransmission in the RVLM, by acting pre‐synaptically to modulate neurotransmitter release, post‐synaptically to modulate receptor function or by modulating the excitability of local interneurons. Therefore, the present study aimed to use blockade of RVLM AT1, VP V1a, OT (in the same receptor subfamily as VP receptors) or ionotropic glutamate receptors to examine whether the pressor effect of [Pyr^1^]apelin‐13 evoked from the RVLM was mediated by angiotensinergic, vasopressinergic, oxytocinergic or glutamatergic neurotransmission. Additionally, because RVLM sympathoexcitatory neurons are tonically inhibited by GABAergic inputs, the effect of blockade of RVLM GABA_A_ receptors on [Pyr^1^]apelin‐13‐induced sympathetic excitation and BP responses within the RVLM was also determined.

## Methods

### Ethical approval

Male Wistar rats (250 g; Charles River, Margate, UK) were housed under a 14:10 h light/dark cycle at 21 ± 2°C and constant relative humidity with *ad libitum* access to standard laboratory chow and water. All experiments were approved by the University of Bristol Animal Welfare and Ethical Review Body and performed in strict accordance with UK Home Office regulations (United Kingdom Home Office Guidelines Scientific Procedures Act of 1986).

### Animal preparation and BP recording

Rats (*n* = 6 per group) were anaesthetized by i.p. injection of sodium pentobarbital (50 mg kg^−1^). All surgical procedures were performed under aseptic conditions under a surgical plane of anaesthesia as indicated by the absence of withdrawal reflex to hindpaw pinch, which was monitored throughout the procedure and additional doses of anaesthetic given if necessary. Rats used in the l‐glutamate experiments were intubated and ventilated with pure O_2_ via a tracheal cannula throughout the experiment (60–70 strokes min^−1^, 1.5 ml; Harvard Apparatus, Cambridge, MA, USA). BP was monitored via a heparin‐saline (1 U ml^−1^) filled catheter (0.8 mm inner diameter; Micro‐Renathane tubing; Braintree Scientific, Braintree, MA, USA) implanted in the left or right femoral artery and connected to a pressure transducer. Pressure signals were amplified and filtered using a NeuroLog System (Digitimer, Welwyn Garden City, UK) and recorded using a CED 1401 data capture system and Spike, version 2.7 (Cambridge Electronic Design, Cambridge, UK). Rats were placed in a stereotaxic frame with the head flexed down (nose bar at −19 mm). The dorsal brainstem was exposed following partial removal of the occipital bone and the dura mater.

### RVLM microinjections

Drug solutions were prepared in accordance with the manufacturers' instructions, diluted to the required concentration (consistent with a large body of literature on the physiological effects of drugs used in the RVLM and other brain regions) (Zhang *et al*. 2009a, b; Huber & Schreihofer 2011; Zhang *et al*. 2014; Du *et al*. 2013; Seyedabadi *et al*. 2002; Kc *et al*. 2002; Mack *et al*. 2002; Lu *et al*. 2015) in normal saline and aliquots stored at −20°C. RVLM injections were made using single‐barrelled micropipettes (1–5 μl calibrated microcapillary tube; Sigma‐Aldrich, Poole, UK), pulled and cut to give a tip diameter of < 100 μm, connected to a manual pressure injection device. The micropipette was aligned with the caudal point of the obex as the reference with the aid of a binocular surgical microscope (M651; Leica Microsystems, Milton Keynes, UK) and the RVLM was targeted using previously established stereotaxic co‐ordinates: 0.18 cm lateral from midline, 0.12 cm rostral and 0.35 cm ventral. Bilateral microinjection of L‐glutamate (1 nmol 100 nl side^−1^; Tocris Bioscience, Bristol, UK) was used to confirm the position of the pipette within the RVLM. The injection volume was determined using the calibration of the micropipette, where 100 nl corresponded to approximately 1 mm of pipette length. Subsequent bilateral microinjections (100 nl side^−1^) were made at the same stereotaxic co‐ordinates that elicited the largest l‐glutamate pressor response (> 20 mmHg). Rats were divided into six experimental groups (*n* = 6 per group) according to the receptor system being targeted (APJ, AT1, VP V1a, OT receptor, ionotropic glutamate receptors, GABA_A_). Successful receptor blockade was tested by recording BP and heart rate (HR) responses to microinjections (100 nl) of the respective agonist for the receptor of interest [[Pyr^1^]‐apelin‐13, 200 pmol (Seyedabadi *et al*. 2002), Bachem, Weil am Rhein, Germany; Ang II, 100 pmol (Du *et al*. 2013), Sigma‐Aldrich; VP, 50 pmol (Kc *et al*. 2002), Bachem, Germany; OT, 50 pmol (Mack *et al*. 2002), Bachem, Germany; l‐glutamate, 1 nmol (Huber & Schreihofer 2011), or muscimol, 2.5 pmol (Lu *et al*. 2015), Tocris Bioscience] before and after microinjection of the respective antagonist [[Ala^13^]apelin‐13, F13A, 2 nmol (Zhang *et al*. 2014), Phoenix Pharmaceuticals, Belmont, CA, USA USA; losartan potassium, 1 nmol (Huber & Schreihofer 2011), Tocris Bioscience; SR 49059, 200 pmol, Sigma‐Aldrich; desGly‐NH_2_, d(CH_2_)_5_[D‐Tyr^2^,Thr^4^]OVT (dOVT), 200 pmol, gift from Professor M. Manning, University of Toledo, Toledo, OH, USA; kynurenic acid, 2.7 nmol (Huber & Schreihofer 2011), Sigma‐Aldrich; or 1(S),9(R)‐(–)‐bicucilline methiodide, 10 pmol (Zhang *et al*. 2009a), Sigma‐Aldrich)]. Microinjections were made in the order: saline; agonist; [Pyr^1^]apelin‐13; antagonist; agonist; [Pyr^1^]apelin‐13; saline. Apelin‐dependent heterodimerization of APJ and AT1 *in vitro* causes negative allosteric regulation of AT1 (Siddiquee *et al*. 2013); therefore, drugs were administered in this order to ensure that BP responses to primary injection of Ang II would be observed. The second injection of [Pyr^1^]apelin‐13 was made no longer than 5 min after the injection of antagonist, assuming no response was observed following the second injection of agonist. To reconfirm micropipette position within the RVLM, a final bilateral injection of l‐glutamate was made after the final injection of [Pyr^1^]apelin‐13. Rats therefore received a total of nine bilateral RVLM microinjections (900 nl RVLM^−1^) over the course of the entire experiment (approximately 2.5 h). A subset of animals received a microinjection of Indian ink (100 nl; 1:10 dilution) at the end of the experiment to confirm the histological location of the micropipette within the RVLM. After the final glutamate and/or bilateral injection of Indian ink, rats were killed using a guillotine. Brains were immediately frozen on dry ice and stored at −80°C. Fresh, frozen sections (40 μm) were cut using a cryostat (CM3050 S; Leica Microsystems) and counterstained with toluidine blue (0.1%). Sections were assessed using a Leica DM IRB microscope and images captured (DC300 F camera; Leica Microsystems).

### Data analysis

BP waveforms from the entire experiment were processed offline using the HRBP, version 8, for Spike 2 to extract systolic, diastolic and mean BP and HR waveforms. Representative APB and HR readings are shown. Data are presented as a change in the physiological variable by subtraction of the post‐microinjection response from the pre‐microinjection baseline for each drug. Spectral analysis of heart rate variability (HRV) and systolic blood pressure variability (SBPV) allows the calculation of power in the very low frequency (VLF: 0–0.3 Hz), low frequency (LF: 0.3–0.8 Hz) and high frequency (HF: 0.8–3.3 Hz) bands (Japundzic‐Zigon 1998; Parati *et al*. 1995). It is known that LF:HF‐HRV and LF‐SBPV can be used as indicative measures of cardiac and vasomotor sympathetic tone, respectively (Lozić *et al*. 2014; Abdala *et al*. 2012); however, we have acknowledged that spectral analysis is an indirect measure of global change in sympathetic activity. Analysis of systolic blood pressure and heart rate was performed using the HRV1 script for Spike 2 to calculate power in the VLF (0–0.3 Hz), LF (0.3–0.8 Hz) and HF (0.8–3.3 Hz) bands of across a 500 s period, including time points before and after microinjection of each drug. The settings used to compute the power spectra were: time constant for DC removal: ±3 s; frequency range of spectra: 0–5.12 Hz; epoch duration: 25 s; FFT size: 64; window type: Hanning.

### Statistical analysis

All data are presented as the mean ± SEM and were tested for statistical significance with a repeated measures one‐way ANOVA followed by Tukey's multiple comparison test using Prism, version 5.0 (Graphpad Software Inc., San Diego, CA, USA). *P* < 0.05 was considered statistically significant between groups (*n* = 6).

## Results

### Verification of microinjection site

**Figure 1:**
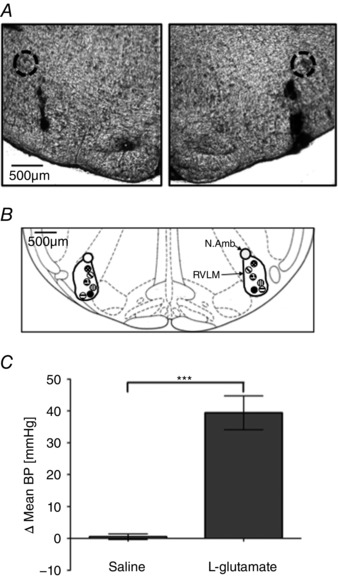
Localization and verification of microinjection sites to the RVLM *A*, representative photomicrographs of toluidine blue stained sections of the rat brainstem with bilateral indian ink (dilution 1:10) microinjected in the position of the RVLM, with the nucleus ambiguus circled for reference. *B*, schematic illustrating the localization of injection sites (matching dots indicate bilateral injection sites), representative of sections from each animal group, determined by examination of the deposition of dye in the brain stem of animals. RVLM and nucleus ambiguus (N. Amb) are indicated. Adapted from Paxinos & Watson (1998 ; figure 68). *C*, change in MABP following bilateral infusion of l‐glutamate, confirming micropipette position within the RVLM. ^***^ *P* < 0.001

Histological confirmation of the microinjection site, and changes observed in MABP with microinjections of l‐glutamate into the RVLM, are shown in Fig. 1 *A* and *C*. Figure 1 *A* shows representative photomicrographs of toluidine blue stained sections of the rat brainstem with bilateral indian ink (dilution 1:10) microinjected in the position of the RVLM. Figure 1 *B* shows a schematic illustrating the localization of injection sites (matching dots indicate bilateral injection sites), representative of sections from each animal group, which were determined by examination of the deposition of dye in the brainstem of animals. At these sites, microinjection of l‐glutamate, as used to assess functionally the position of the micropipette in the RVLM at the beginning of each experiment (*n* = 36), caused a significant increase in MABP compared to saline (39 ± 5 mmHg compared to1 ± 1 mmHg; *P* < 0.001) (Fig. 1 *C*).

### Effects of F13A microinjection into the RVLM on [Pyr^1^]apelin‐13‐mediated BP and SNA

**Figure 2:**
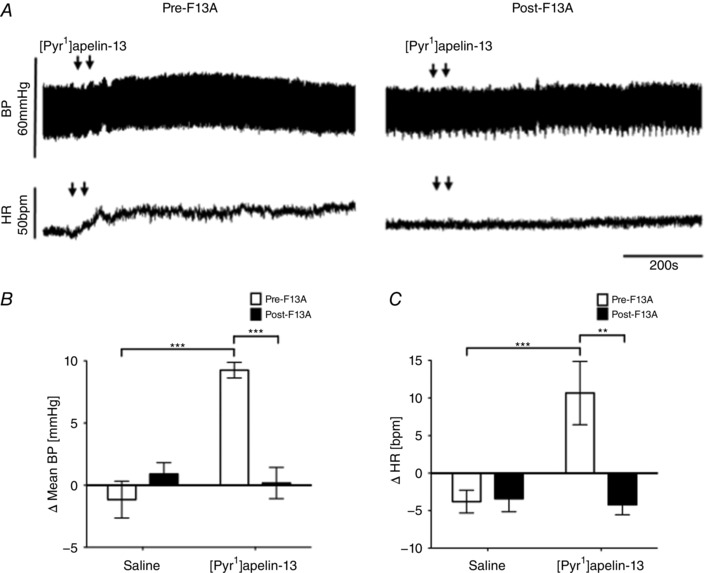
Effects of APJ agonist and antagonist microinjection into the RVLM on blood pressure and heart rate *A*, raw BP and HR traces showing representative responses following the bilateral microinjection of [Pyr^1^]apelin‐13 (200 pmol 100 nl side^−1^) before and after the injection of the APJ antagonist F13A (2 nmol 100 nl side^−1^). Arrows mark the bilateral injection of [Pyr^1^]apelin‐13. *B* and *C*, changes in MABP (*B*) and HR (*C*) following bilateral microinjection of saline vehicle control and [Pyr^1^]apelin‐13 before (white bars) and after (black bars) administration of F13A. *B* and *C*, data are shown as the mean ± SEM (*n* = 6). ^**^ *P* < 0.01, ^***^ *P* < 0.001. Scale bars for BP, HR and time are indicated.

An ABP and HR recording following microinjection of [Pyr^1^]apelin‐13 pre‐ and post‐F13A microinjection into the RVLM is shown in Fig. 2 *A*. The mean basal BP and HR, calculated across rats from all groups (*n* = 36), were 84 ± 3 mmHg and 350 ± 9 beats min^–1^, respectively. Microinjection of [Pyr^1^]apelin‐13 (200 pmol) in the RVLM increased MABP (9 ± 1 mmHg; *P* < 0.001) (Fig. 2 *B*) and HR (11 ± 4 beats min^–1^; *P* < 0.001) (Fig. 2 *C*) compared to saline‐injected controls (−1 ± 2 mmHg and −6 ± 2 beats min^–1^) (Fig. 2 *B* and *C*). The effect peaked at 463 ± 64 s post injection, and had a duration of 997 ± 103 s (Fig. 2 *A*). Microinjection of [Pyr^1^]apelin‐13 into areas surrounding the RVLM, including the facial nucleus, rubrospinal tract and gigantocellular nucleus, had no significant effect on MABP or HR. LF‐SBP and LF:HF HR variability spectral domains increased after [Pyr^1^]apelin‐13 microinjection (13.9 ± 4.3% and 0.73 ± 0.33% total power) (Tables 1 and 2), indicating an increase in sympathetic drive to the vasculature and heart respectively, albeit indirectly. Microinjection of the APJ antagonist F13A into the RVLM abolished the [Pyr^1^]apelin‐13‐mediated increases in MABP (0 ± 1 mmHg; *P* < 0.01), HR (−4 ± 1 beats min^–1^; *P* < 0.05) and sympathetic vasomotor activation (−0.6 ± 1.5% and −0.05 ± 0.04% total power; *P* < 0.01 and *P* < 0.05) (Fig. 2 *A* and *C* and Tables 1 and 2). Administration of F13A alone had no significant effect on BP or HR (*P *> 0.05) (Table 3) suggesting an absence of endogenous apelinergic drive in the RVLM of anaesthetized rats.

**Table 1:** Effects of agonists injected into the RVLM on LF‐SBP

	Δ LF‐SBP (% total power)	
	Pre‐F13A	Post‐F13A
Saline	−2.2 ± 2.0	0.4 ± 1.3
[Pyr^1^]apelin‐13	13.9 ± 4.3***	−0.6 ± 1.5††
	Pre‐losartan	Post‐losartan
Saline	3.0 ± 2.0	1.0 ± 1.6
[Pyr^1^]apelin‐13	11.5 ± 4.0*	10.4 ± 2.0*
Ang II	13.9 ± 4.3*	1.0 ± 1.5‡
	Pre‐SR 49059	Post‐SR 49059
Saline	1.3 ± 1.4	1.3 ± 1.5
DMSO	1.1 ± 1.3	0.2 ± 1.1
[Pyr^1^]apelin‐13	19.2 ± 3.8***, +++	1.1 ± 1.1†††
VP	16.9 ± 5.5***, +++	1.1 ± 1.8‡‡
	Pre‐dOVT	Post‐dOVT
Saline	2.8 ± 1.8	1.3 ± 0.6
[Pyr^1^]apelin‐13	19.2 ± 1.4*	17.2 ± 3.8
OT	35.6 ± 7.5***	2.7 ± 1.4‡‡‡
	Pre‐kynurenic acid	Post‐kynurenic acid
Saline	0.8 ± 2.0	0.8 ± 1.7
[Pyr^1^]apelin‐13	9.8 ± 1.7*	8.5 ± 1.1*
Glutamate	23.5 ± 8.1**	−0.8 ± 2.0‡‡
	Pre‐bicuculline	Post‐bicuculline
Saline	0.2 ± 0.3	0.1 ± 0.2
[Pyr^1^]apelin‐13	7.3 ± 1.1***	4.4 ± 0.8***
Muscimol	−8.5 ± 1.2***	−0.7 ± 1.0‡‡‡

^*^ *P* < 0.05, ^**^ *P* < 0.01, ^***^ *P* < 0.001 *vs*. saline. ^+^ *P* < 0.05, ^++^ *P* < 0.01, ^+++^ *P* < 0.001 *vs*. DMSO. ^†^ *P* < 0.05, ^††^ *P* < 0.01, ^†††^ *P* < 0.001 [Pyr^1^]apelin‐13 pre‐ *vs*. [Pyr^1^]apelin‐13 post‐antagonist. ^‡^ *P* < 0.05, ^‡‡^ *P* < 0.01, ^‡‡‡^ *P* < 0.001 Agonist pre‐ *vs*. agonist post‐antagonist.

**Table 2:** Effects of agonists injected into the RVLM on LF:HF HR

	Δ LF:HF HR	
	Pre‐F13A	Post‐F13A
Saline	0.05 ± 0.03	−0.02 ± 0.01
[Pyr^1^]apelin‐13	0.73 ± 0.33*	−0.05 ± 0.04†
	Pre‐losartan	Post‐losartan
Saline	0.01 ± 0.01	−0.03 ± 0.03
[Pyr^1^]apelin‐13	0.37 ± 0.15*	0.27 ± 0.10
Ang II	−0.17 ± 0.05**	−0.01 ± 0.01‡‡
	Pre‐SR 49059	Post‐SR 49059
Saline	−0.04 ± 0.03	−0.02 ± 0.01
DMSO	0.02 ± 0.02	−0.01 ± 0.02
[Pyr^1^]apelin‐13	0.38 ± 0.11***, +++	0.01 ± 0.01†††
VP	0.26 ± 0.07**, +	−0.02 ± 0.01‡‡
	Pre‐dOVT	Post‐dOVT
Saline	0.01 ± 0.01	−0.02 ± 0.03
[Pyr^1^]apelin‐13	0.49 ± 0.15*	0.52 ± 0.13**
OT	0.38 ± 0.18	0.07 ± 0.04
	Pre‐kynurenic acid	Post‐kynurenic acid
Saline	0.02 ± 0.02	−0.01 ± 0.01
[Pyr^1^]apelin‐13	0.17 ± 0.03*	0.15 ± 0.04**
Glutamate	0.42 ± 0.11*	0.02 ± 0.10‡
	Pre‐bicuculline	Post‐bicuculline
Saline	0.02 ± 0.01	−0.02 ± 0.02
[Pyr^1^]apelin‐13	0.12 ± 0.01	0.17 ± 0.04*
Muscimol	0.14 ± 0.04*	−0.02 ± 0.04‡‡

^*^ *P* < 0.05, ^**^ *P* < 0.01, ^***^ *P* < 0.001 *vs*. saline. ^+^ *P* < 0.05, ^++^ *P* < 0.01, ^+++^ *P* < 0.001 *vs*. DMSO. ^†^ *P* < 0.05, ^††^ *P* < 0.01, ^†††^ *P* < 0.001 [Pyr^1^]apelin‐13 pre‐ *vs*. [Pyr^1^]apelin‐13 post‐antagonist. ^‡^ *P* < 0.05, ^‡‡^ *P* < 0.01, ^‡‡‡^ *P* < 0.001 Agonist pre‐ *vs*. agonist post‐antagonist.

**Table 3:** Effects of receptor antagonists microinjected alone into the RVLM on BP and HR

	ΔMABP (mmHg, *n* = 6)		ΔHR (beats min^–1^, *n* = 6)	
	Saline	Antagonist	Saline	Antagonist
F13A (2 nmol)	−1 ± 2	−1 ± 4	−6 ± 2	2 ± 4
Kynurenic acid (2.7 nmol)	−0 ± 2	1 ± 1	−4 ± 6	−4 ± 7
Bicuculline (10 pmol)	2 ± 2	6 ± 3	−1 ± 0	−6 ± 3
Losartan (1 nmol)	0 ± 1	3 ± 2	−6 ± 3	−3 ± 5
SR49059 (0.2 nmol)	0 ± 1	−1 ± 2	−3 ± 5	−3 ± 3
dOVT (0.2 nmol)	−1 ± 1	0 ± 1	2 ± 2	2 ± 3

Values are the mean ± SEM.

### Effect of blockade of angiotensin II type AT1 receptors on [Pyr^1^]apelin‐13‐induced BP and sympathetic excitation within the RVLM

**Figure 3:**
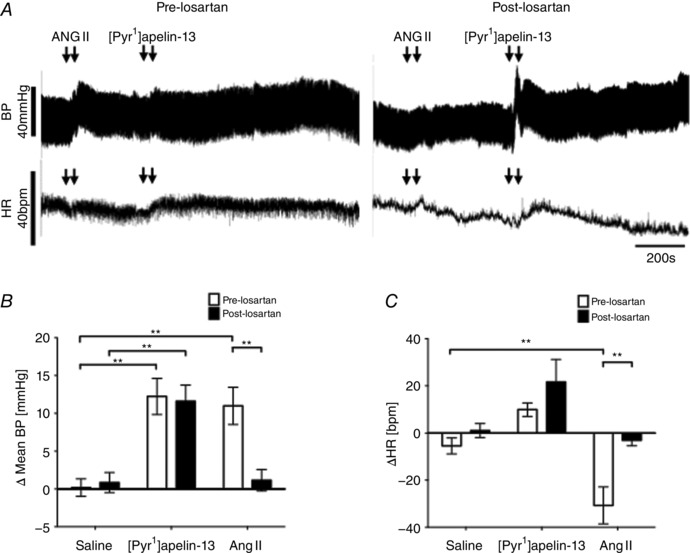
Effect of blockade of RVLM Ang II AT1 receptors on [Pyr^1^]apelin‐13‐induced cardiovascular responses *A*, raw BP and HR traces showing representative responses following the bilateral microinjection of Ang II (100 pmol 100 nl^−1^) and [Pyr^1^]apelin‐13 (200 pmol 100 nl side^−1^) before and after the injection of the AT1 receptor antagonist, losartan (1 nmol 100 nl side^−1^). Arrows mark the times of bilateral injections. *B* and *C*, changes in MABP (*B*) and HR (*C*) following bilateral microinjection of saline vehicle control, [Pyr^1^]apelin‐13 and Ang II before (white bars) and after (black bars) administration of losartan. *B* and *C*, data are shown as the mean ± SEM (*n* = 6). ^**^ *P* < 0.01. Scale bars for BP, HR and time are indicated.

Figure 3 *A* shows the effects of the AT1 receptor antagonist losartan on BP and HR responses elicited by [Pyr^1^]apelin‐13 and Ang II injection. Bilateral microinjection of [Pyr^1^]apelin‐13 in the RVLM induced an increase in BP (12 ± 2 mmHg; *P* < 0.01) (Fig. 3 *B*) and HR (10 ± 3 beats min^–1^) (Fig. 3 *C*) compared to saline‐injected controls (0 ± 1 mmHg and –6 ± 3 beats min^–1^) (Fig. 3 *B* and *C*) and these responses were not significantly affected by prior microinjection of losartan into the RVLM (*P *> 0.05) (Fig. 3 *B* and *C*). Bilateral microinjection of Ang II into the RVLM increased BP (11 ± 3 mmHg; *P* < 0.01) (Fig. 3 *B*) and decreased HR (−31 ± 8 beats min^–1^; *P* < 0.01) (Fig. 3 *C*) compared to saline‐injected controls; these responses were blocked by pretreatment with losartan (2 ± 1 mmHg and −1 ± 2 beats min^–1^; *P* < 0.01 and *P* < 0.01) (Fig. 3 *B* and *C*). Administration of losartan alone did not change basal BP and HR parameters (Table 3).

Spectral analysis of SBP and HR variability showed that pre‐injection with losartan had no effect on the increases in SBP and HR components seen after bilateral injection of [Pyr^1^]apelin‐13 (*P *> 0.05) (Tables 1 and 2). However microinjection of Ang II raised LF‐SBP and decreased the LF:HF HRV ratio compared to saline injected controls and pre‐administration of losartan into the RVLM abolished these responses (Tables 1 and 2).

### Cardiovascular effects of [Pyr^1^]apelin‐13 in the RVLM after blockade of VP V1a or OT receptors

**Figure 4:**
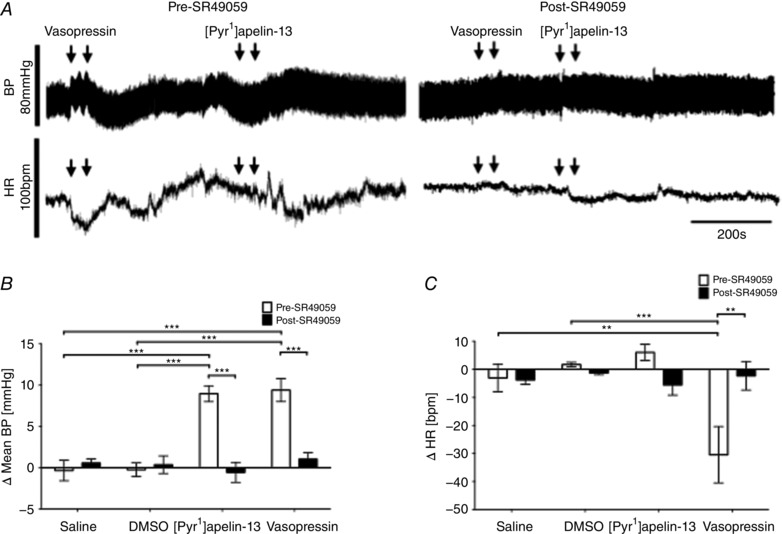
Cardiovascular effects of [Pyr^1^]apelin‐13 in the RVLM after blockade of VP V1a receptors *A*, raw BP and HR traces showing representative responses following the bilateral microinjection of VP (50 pmol 100 nl side^−1^) and [Pyr^1^]apelin‐13 (200 pmol 100 nl side^−1^) before and after the injection of the VP V1a receptor antagonist, SR 49059 (200 pmol 100 nl side^−1^). Arrows mark the times of bilateral injections. *B* and *C*, changes in MABP (*B*) and HR (*C*) following bilateral microinjection of saline vehicle control, [Pyr^1^]apelin‐13 and VP before (white bars) and after (black bars) administration of SR 49059. *B* and *C*, data are shown as the mean ± SEM (*n* = 6). ^**^ *P* < 0.01, ^***^ *P* < 0.001. Scale bars for BP, HR and time are indicated.

The effects of the VP V1a receptor antagonist SR 49059 on BP and HR responses to [Pyr^1^]apelin‐13 and VP in the RVLM are shown in Fig. 4 *A*. The BP (9 ± 1 mmHg; *P* < 0.001) (Fig. 4 *B*) and HR (6 ± 3 beats min^–1^) (Fig. 4 *C*) responses to bilateral microinjection of [Pyr^1^]apelin‐13 were effectively abolished after pretreatment with SR 49059 (−1 ± 1 mmHg and −6 ± 4 beats min^–1^; *P* < 0.001) (Fig. 3 *B* and *C*). As expected, the BP and HR responses to bilateral microinjection of VP (9 ± 1 mmHg and −30 ± 10 beats min^–1^; *P* < 0.001 and *P* < 0.001) (Fig. 3 *B* and *C*) were significantly decreased after pretreatment with SR 49059 (1 ± 1 mmHg and −2 ± 5 beats min^–1^; *P* < 0.001 and *P* < 0.01) (Fig. 4 *B* and *C*). The response latency and duration of BP responses to VP were 64 ± 6 s and 346 ± 25 s respectively, which is significantly shorter than that seen with [Pyr^1^]apelin‐13 (*P* < 0.001) (Fig. 4 *A*; see also Fig. 2 *A*). Bilateral microinjection of SR 49059 alone had no significant effect on ABP and HR (*P *> 0.05) (Table 3).

**Figure 5:**
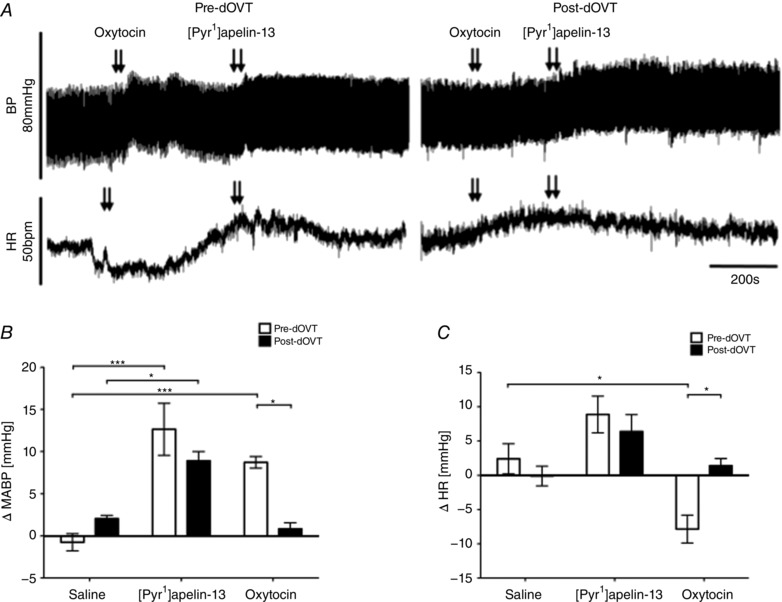
Effect of RVLM oxytocin receptor antagonist microinjection on [Pyr^1^]apelin‐13‐induced cardiovascular responses *A*, raw BP and HR traces showing representative responses following the bilateral microinjection of OT (50 pmol 100 nl side^−1^) and [Pyr^1^]apelin‐13 (200 pmol 100 nl side^−1^) before and after the injection of the OT receptor antagonist, dOVT (200 pmol 100 nl side^−1^). Arrows mark the times of bilateral injections. *B* and *C*, changes in MABP (*B*) and HR (*C*) following bilateral microinjection of saline vehicle control, [Pyr^1^]apelin‐13 and OT before (white bars) and after (black bars) administration of dOVT. *B* and *C*, data are shown as the mean ± SEM (*n* = 6). ^*^ *P* < 0.05, ^***^ *P* < 0.001. Scale bars for BP, HR and time are indicated.

ABP and HR recordings depicting the effects of blockade of OT receptors with dOVT on BP and HR responses to [Pyr^1^]apelin‐13 and OT pre‐ and post‐antagonist microinjection are shown in Fig. 5 *A*. Bilateral microinjection of OT increased BP and HR (9 ± 1 mmHg and −8 ± 2 beats min^–1^; *P* < 0.001 and *P* < 0.05) (Fig. 5 *B* and *C*) and these responses were abolished after pretreatment with dOVT (1 ± 1 mmHg and 1 ± 1 beats min^–1^; *P* < 0.01 and *P* < 0.05) (Fig. 5 *B* and *C*). The BP (13 ± 3 mmHg; *P* < 0.001) (Fig. 5 *B*) and HR (9 ± 3 beats min^–1^) (Fig. 5 *C*) responses to bilateral microinjection of [Pyr^1^]apelin‐13 were unaffected by pretreatment with dOVT (9 ± 1 mmHg and 6 ± 2 beats min^–1^) (Fig. 5 *B* and *C*). Bilateral microinjection of dOVT alone had no significant effect on ABP and HR (*P *> 0.05) (Table 3).

[Pyr^1^]apelin‐13 microinjection‐induced increases in the LF‐SBP band power and LF:HF‐HRV ratio were reduced to baseline saline‐injected levels by pre‐injection of SR 49059 (*P *< 0.001) (Tables 1 and 2) but not by pre‐injection of dOVT (Tables 1 and 2). Pre‐treatment with either SR 49059 or dOVT, however, abolished the cardiovascular short‐term variability responses seen after bilateral microinjection of VP or OT, respectively (Tables 1 and 2).

### Effect of blockade of ionotropic glutamate receptors on [Pyr^1^]apelin‐13‐induced BP and sympathetic excitation within the RVLM

**Figure 6:**
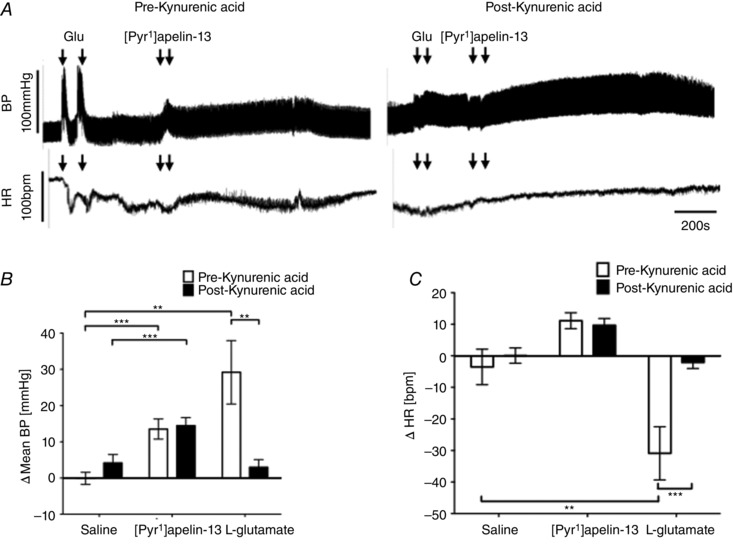
Effect of blockade of RVLM ionotropic glutamate receptors on [Pyr^1^]apelin‐13‐induced cardiovascular responses *A*, raw BP and HR traces showing representative responses following the bilateral microinjection of L‐glutamate (1 nmol 100 nl side^−1^) and [Pyr^1^]apelin‐13 (200 pmol 100 nl side^−1^) before and after the injection of the ionotropic glutamate receptor antagonist, kynurenic acid (2.7 nmol 100 nl side^−1^). Arrows mark the times of bilateral injections. *B* and *C*, changes in MABP (*B*) and HR (*C*) following bilateral microinjection of saline vehicle control, [Pyr^1^]apelin‐13 and L‐glutamate before (white bars) and after (black bars) administration of kynurenic acid. *B* and *C*, data are shown as the mean ± SEM (*n* = 6). ^**^ *P* < 0.01, ^***^ *P* < 0.001. Scale bars for BP, HR and time are indicated.

To determine whether glutamatergic activation of the RVLM contributes to [Pyr^1^]apelin‐13‐mediated changes in BP, HR and sympathetic drive, glutamatergic inputs to the RVLM were blocked by bilateral microinjections of kynurenate. The BP and HR responses to [Pyr^1^]apelin‐13 and l‐glutamate before and after injection of kynurenate are shown in Fig. 6 *A*. Bilateral injection of [Pyr^1^]apelin‐13 into the RVLM increased BP (14 ± 3 mmHg; *P* < 0.001) (Fig. 6 *B*) and HR (11 ± 3 beats min^–1^) (Fig. 6 *C*) compared to saline‐injected controls (0 ± 2 mmHg and −4 ± 6 beats min^–1^) (Fig. 6 *B* and *C*). The pressor and tachycardic effects of [Pyr^1^]apelin‐13 injection were not significantly affected by prior microinjection of kynurenate into the RVLM (15 ± 2 mmHg and 10 ± 2 beats min^–1^; *P *> 0.05 and *P *> 0.05) (Fig. 6 *B* and *C*). To confirm effective blockade of ionotropic glutamate receptors, BP and HR responses following bilateral injection of l‐glutamate were compared before and after administration of kynurenate (BP: *P* < 0.01; HR: *P* < 0.001) (Fig. 6 *B* and *C*). Administration of kynurenate alone resulted in minimal changes to BP and HR (Table 3).

Following microinjection of kynurenate into the RVLM, the LF‐SBP and LF:HF‐HRV responses associated with [Pyr^1^]apelin‐13 injection were not significantly different compared to pre‐antagonist responses (*P *> 0.05) (Tables 1 and 2 respectively). By contrast, increases in LF‐SBP and LF:HF‐HRV ratio associated with l‐glutamate injection were attenuated following kynurenate injection to the RVLM (Tables 1 and 2).

### Effect of blockade of GABA_A_ receptors on [Pyr^1^]apelin‐13‐induced BP and sympathetic excitation within the RVLM

**Figure 7:**
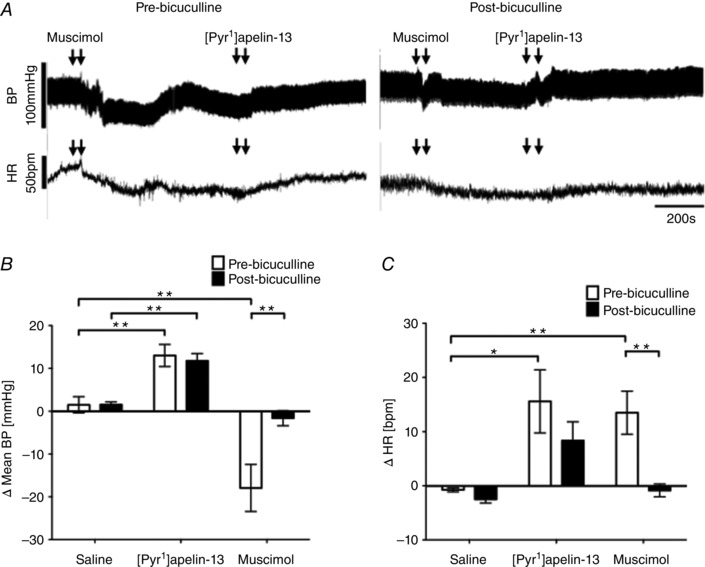
Cardiovascular effects of [Pyr^1^]apelin‐13 in the RVLM after blockade of GABA_A_ receptors *A*, raw BP and HR traces showing representative responses following the bilateral microinjection of muscimol (2.5 pmol 100 nl side^−1^) and [Pyr^1^]apelin‐13 (200 pmol 100 nl side^−1^) before and after the injection of the GABA_A_ receptor antagonist, bicuculline (10 pmol 100 nl side^−1^). Arrows mark the times of bilateral injections. *B* and *C*, changes in MABP (*B*) and HR (*C*) following bilateral microinjection of saline vehicle control, [Pyr^1^]apelin‐13 and muscimol before (white bars) and after (black bars) administration of bicuculline. *B* and *C*, data are shown as the mean ± SEM (*n* = 6). ^*^ *P* < 0.05, ^**^ *P* < 0.01.

ABP and HR recordings of the effects of blockade of GABA_A_ receptors with bicuculline on BP and HR responses to [Pyr^1^]apelin‐13 and muscimol pre‐ and post‐antagonist microinjection are shown in Fig. 7 *A*. Pre‐treatment with bicuculline did not significantly change the increases in MABP (13 ± 3 mmHg; *P* < 0.01) (Fig. 7 *B*) or HR (16 ± 6 beats min^–1^; *P* < 0.05) (Fig. 7 *C*) induced by the microinjection of [Pyr^1^]apelin‐13 into the RVLM compared to saline‐injected controls (−1 ± 1 mmHg and −3 ± 1 beats min^–1^) (Fig. 7 *B* and *C*). Prior to bicuculline microinjection, microinjection of muscimol into the RVLM reduced baseline MABP and increased HR compared to saline‐injected controls (*P* < 0.01) Fig. 7 *B* and *C*) and these BP and HR responses were abrogated after blockade of GABA_A_ receptors in the RVLM (*P* < 0.01) (Fig. 7 *B* and *C*). Bicuculline injection alone caused small changes in ABP and HR; however, these were not significantly different compared to saline (*P *> 0.05) (Table 3).

Similarly, the increases in LF‐SBP band power and the LF:HF‐HRV ratio seen after [Pyr^1^]apelin‐13 microinjection into the RVLM compared to saline pretreatment were unaffected by pre‐injection of bicuculline (*P *> 0.05) (Tables 1 and 2). Pre‐treatment with this antagonist however reduced the LF‐SBP band power and the LF:HF‐HRV ratio responses seen after bilateral microinjection of muscimol (Tables 1 and 2).

## Discussion

In the present study, we show that the pressor effect evoked in the RVLM by bilateral microinjection of [Pyr^1^]apelin‐13 is dependent on VP V1a receptor activation. On the other hand, blockade of AT1, OT, ionotropic glutamate or GABA_A_ receptors in the RVLM has no effect on the pressor and sympathoexcitatory response evoked by [Pyr^1^]apelin‐13.

Bilateral microinjection of [Pyr^1^]apelin‐13 into the RVLM of Wistar rats increased BP and sympathetic vasomotor tone (as indirectly measured by spectral analysis of BP), which is consistent with previous studies in normotensive WKY (Zhang *et al*. 2009b) and Sprague–Dawley (Seyedabadi *et al*. 2002) rats. The rise in [Pyr^1^]apelin‐13‐mediated BP was associated with an increase in the low frequency spectra of systolic BP, indicative of sympathetic vasomotor activation, and an increase in LF:HF HRV, a measure of sympathovagal balance, suggesting a predominantly sympathetic influence (Malliani *et al*. 1991). There was no obvious autonomically mediated chronopic effect following microinjection of apelin; thus, vagal drive to the heart may not be influenced by the apelin‐mediated pressor response. Inconsistent effects of i.v. injected apelin on HR have been reported previously, showing either an increase (Mitra *et al*. 2006) or no effect (Reaux *et al*. 2001). Pre‐treatment with the specific APJ antagonist, F13A (Lee *et al*. 2005), abolished the [Pyr^1^]apelin‐13‐induced pressor activity and sympathetic outflow; however, the administration of F13A alone did not produce a depressor response, suggesting that, if apelin is endogenously released, it is not associated with tonic neural control of resting cardiovascular activity in the RVLM of normotensive rats.

In the mouse, apelin‐13 has been shown to alter the consolidation of passive avoidance learning and to exhibit anxiolytic‐like activity via multiple peripheral mechanisms, including α‐adrenergic, 5‐HT_2_ serotonergic, cholinergic, dopaminergic and GABA_A_ergic systems (Telegdy *et al*. 2013; Telegdy & Jászberényi 2014). Because these findings suggest that apelin may have a role as a modulatory neurotransmitter, we proceeded to explore the possible mechanisms by which [Pyr^1^]apelin‐13 in the RVLM affects MABP and SNA. We evaluated the role of Ang II, VP, glutamate and GABA neurotransmission on the cardiovascular effects mediated by bilateral RVLM [Pyr^1^]apelin‐13 microinjection. Ang II, acting via the AT1 receptor expressed in the RVLM, is known to function as an excitatory modulator increasing BP and sympathetic outflow (Mayorov & Head 2003a), as well as increasing the excitability of spinally projecting PVN neurons by attenuation of GABAergic synaptic inputs (Li *et al*. 2003), whereas VP is a potent vasoconstrictor and the pressor responses of excitatory amino acids injected into the brain have been shown to be mediated via VP release (Pizzirusso *et al*. 1998). Additionally l‐glutamate and GABA receptors play important roles in the synaptic regulation of RVLM central pressor responses (Dampney 1994). l‐glutamate regulates neuron excitability, increasing BP and SNA (Guyenet 2006) and enhancing the sympathetic baroreflex (Mayorov & Head 2003b), whereas tonic inhibition of vasopressor neurons in the RVLM by endogenous GABA is well established (Heesch *et al*. 2006) and may play a role in modifying the responsiveness of these neurones to the effects of sympathoexcitatory neurotransmitters.

We show that bilateral microinjection of the selective antagonist losartan to block AT1 receptors in the RVLM does not affect [Pyr^1^]apelin‐13‐mediated increases in BP or SNA in normotensive animals. This suggests that Ang II does not mediate the sympathoexcitatory response to [Pyr^1^]apelin‐13 in this region. The absence of changes in BP and sympathetic outflow seen in the present study is in accordance with previous studies where i.c.v. or PVN injection of the AT1 receptor antagonists CV‐11974 or losartan, respectively, were ineffective at preventing the BP and SNA effects of i.c.v. (Kagiyama *et al*. 2005) or PVN‐ (Zhang *et al*. 2014) injected apelin. Thus, despite APJ sharing highest sequence homology with the AT1 receptor (O'Dowd *et al*. 1993) and the fact that these two receptors appear to heterodimerize, at least *in vitro* (Siddiquee *et al*. 2013), the cardiovascular effects of central apelin do not appear to be mediated via AT1 receptors in normotensive animals.

The apelin–APJ system may also act indirectly via regulation of vasopressinergic neuron activity to affect vascular tone. VP neurons project from the PVN to the RVLM where V1a receptor immunoreactivity is established in RVLM‐spinal vasomotor neurons (Kc *et al*. 2010) and a combination of actions of PVN‐induced VP release in the NTS and RVLM has been postulated to elevate arterial BP (Vallejo *et al*. 1984; Sun & Guyenet 1989). We therefore investigated whether the apelin‐APJ system may act via VP to affect cardiovascular responses in the RVLM. We show that bilateral microinjection of VP into the RVLM of Wistar rats increases BP and sympathetic vasomotor tone, and decreases HR; responses that are abolished after microinjection of the VP V1a antagonist SR 49059 in the RVLM. Importantly, microinjection SR 49059 is also effective in blocking the [Pyr^1^]apelin‐13‐induced increases in MABP, HR and sympathetic tone, suggesting that [Pyr^1^]apelin‐13 acting in the RVLM increases BP and vasomotor tone via a vasopressinergic mechanism. As found for apelin, inconsistent HR responses have been reported previously following the microinjection of VP into the RVLM, where variable and non‐dose‐related changes are observed in the RVLM (Gomez *et al*. 1993) and NTS (Lawrence & Jarrott, 1995), whereas topical application of VP to the ventral surface of the RVLM does not result in consistent or significant tachycardia (Andreatta‐Van Leyen *et al*. 1990). Therefore, it is difficult to draw conclusions from the results of the present study regarding the interplay of apelin and VP in the control of HR via the RVLM.

V1a and OT receptors appear to be differentially distributed within the RVLM, with VP V1a receptors expressed throughout the rostro‐caudal axis (Kc *et al*. 2010) and OT receptors confined to a small population of neurons in the pre‐Botzinger complex (Mack *et al*. 2002). Whether any colocalization of the two receptors exists, or whether there is functional cross‐talk between the two receptor systems in the RVLM, is not known. However, given the relatively high affinity of OT for the VP V1a receptor (Chini & Manning 2007), OT may also be active in modulating BP via RVLM VP V1a receptors. Therefore, we investigated the effect of bilateral microinjection of OT in the RVLM on BP responses. OT microinjection increased MABP and HR in the RVLM compared to saline‐injected controls; responses that were completely abolished after pretreatment with the highly specific OT receptor antagonist dOVT (Manning *et al*. 2012), indicating that OT does not modulate BP via RVLM VP V1a receptors at the ligand doses used in the present study. Furthermore, cardiovascular responses to bilateral microinjection of [Pyr^1^]apelin‐13 were not affected by pre‐treatment with dOVT, suggesting that the pressor action of [Pyr^1^]apelin‐13 in the RVLM normotensive rats is not mediated via OT receptors. Experiments performed in rats in which OT has been knocked down, or in OT knockout mice, may clarify which neurohypophysial hormone is primarily active in the cardiovascular actions mediated via the RVLM.

The precise nature of the interaction between APJ and V1a receptors at the level of the RVLM is not known; however, there is interplay between the apelinergic and vasopressinergic systems in the hypothalamus where a cross‐regulation of endogenous levels of apelin and VP has been described (Reaux‐Le Goazigo *et al*. 2004), as well as in the renal collecting duct where cross‐talk between APJ‐ and VP V1a receptor‐mediated signalling has been proposed (Hus‐Citharel *et al*. 2014). Additionally, we have shown that [Pyr^1^]apelin‐13 increases the firing rate of VP neurons in the isolated SON; such activation invariably correlates with increased VP terminal secretion that may be mediated in part through apelin‐induced inhibition of dendritic VP release within the SON (Tobin *et al*. 2008). Such an interaction, which may involve receptor dimerization as is seen in other GPCR systems (Milligan 2009), may be explored in ongoing studies in *in vitro* systems with cells co‐expressing both receptors or using bioluminescence or fluorescence resonance energy transfer to monitor protein–protein interactions.

Primary neurotransmitters also implicated in excitatory and inhibitory synaptic inputs to sympathoexcitatory RVLM neurons are glutamate and GABA, mediated by ionotropic glutamate and GABA_A_ receptors, respectively (Sun & Guyenet 1985, Sun & Guyenet 1986). A recent report has demonstrated that blockade of NMDA and non‐NMDA glutamate receptors in the PVN attenuates the pressor and sympathetic activity response to apelin‐13 in the PVN in SHR (Zhang *et al*. 2014). The contrasting data of the present study, where no effect on [Pyr^1^]apelin‐13‐mediated increases in BP or SNA is seen after blockade of ionotropic glutamate receptors, may reflect the region studied (RVLM *vs*. PVN) and/or the physiological condition of the animals (normotensive *vs*. SHR). Altered synaptic transmission has been shown in cardiovascular regulatory centres in hypertension models (Wang *et al*. 2009) and both apelin (Zhang *et al*. 2009b) and glutamate (Li *et al*. 2014) receptors are upregulated in the RVLM of SHR, a model of essential hypertension that has an increased basal vasomotor tone. Thus, there may be a shift in the balance of inhibitory and excitatory inputs in SHR brain regions to produce variable physiological changes not seen in the normotensive state. Additionally, blockade of GABA_A_ receptors had no effect on baseline BP in the RVLM or on the pressor and sympathoexcitatory response evoked by [Pyr^1^]apelin‐13, suggesting that the mechanism by which apelin excites RVLM neurons is independent of GABAergic neurotransmission.

**Figure 8:**
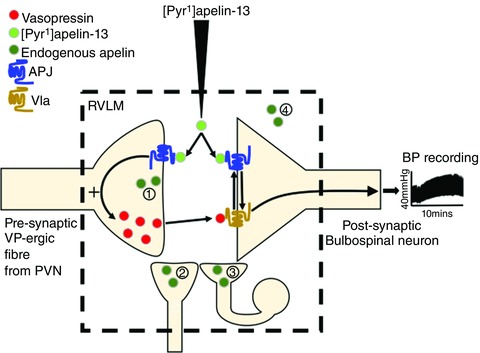
Schematic interpretation of VP V1a receptor mediation of the central hypertensive effect of [Pyr^1^]apelin‐13 [Pyr^1^]apelin‐13 injection, acting via APJ located on presynaptic VP fibres within the RVLM, induces the release of VP with consequent pressor effects. Additionally, APJ located on the postsynaptic membrane of RVLM bulbospinal neurons may interact with the VP V1a receptor (e.g. receptor dimerization) to modulate agonist induced pressor effects. VP release from PVN‐RVLM projecting fibres could also be affected by the presence of presynaptic VP V1a receptors. Potential sources of endogenous apelin are indicated (1) to (4). Apelin could (1) be co‐localized with VP in PVN‐RVLM projecting fibres (apelin‐immunoreactive cell bodies have been described in the magnocellular region of the PVN (Reaux *et al*. 2001)); (2) be present in non‐VP‐ergic fibres projecting to the RVLM; (3) be found in (and released from) local neurons within the RVLM neuronal network (the apelin gene is transcribed in the RVLM (Zhang *et al*. 2009b) and our unpublished observations); and/or (4) reach the RVLM via volume transmission. [Color figure can be viewed at wileyonlinelibrary.com]

Conceivably, [Pyr^1^]apelin‐13 injection, acting via APJ located on VP fibres within the RVLM, induces the release of VP with consequent pressor effects; it is also possible that APJ cross‐talks with the VP V1a receptor on RVLM neuronal cell bodies to modulate agonist‐induced pressor effects (Fig. 8). The elevation in BP is more prolonged when injecting [Pyr^1^]apelin‐13 compared to VP at the same RVLM site. Factors that may contribute to this ‘longer’ time course may include multiple actions where apelin may act at different locations within the RVLM (e.g. different cell/fibre populations) with varying pharmacokinetics. There is evidence for functional heterogeneity in apelin electrophysiological responses (e.g. mainly depolarization, as well as hyperpolarization in some neurons) in other brain regions such as the arcuate nucleus (Lee *et al*. 2015) and subfornical organ (Dai *et al*. 2013). This proposed system may be indicative of a complex mechanism in controlling the physiological functions of endogenous apelin that may be important in a hypertensive disease state, in which apelin secretion increases BP by inducing VP release. Further studies are needed to determine whether there are other vasoactive peptides with which apelin and APJ may interact, as well as the specific mechanisms involved in the regulation of [Pyr^1^]apelin‐13‐mediated cardiovascular control within the RVLM.

In conclusion, the results of the present study confirm that microinjection of [Pyr^1^]apelin‐13 into the RVLM increases mean arterial pressure and SNA, and that these effects are mediated via APJ, suggesting that this peptide system may play an important role in cardiovascular regulation at the level of the RVLM in specific physiological situations and should be considered as a potential target for therapeutic strategies that are used to reverse hypertensive pathology. We show that [Pyr^1^]apelin‐13 acts as a modulating neurotransmitter in the normotensive rat RVLM to facilitate synaptic transmission and that [Pyr^1^]apelin‐13‐induced sympathoexcitation acts via the VP V1a receptor to affect vascular tone, but is independent of AT1, OT, ionotropic glutamate and GABA_A_ receptors.

## Additional information

### Competing interests

The authors declare they have no competing interests.

### Author contributions

A‐MO'C, SJL and JFRP were responsible for acquisition of funding. A‐MO'C, JFRP and SJL contributed to the conception and design of the research. PRG and LEH performed the experiments. A‐MO'C, JFRP, SJL and PRG interpreted the results of the experiments. A‐MO'C and PRG drafted the manuscript. A‐MO'C, JFRP, SJL, PRG and LEH edited and revised the manuscript. All authors have approved the final version of the manuscript and agree to be accountable for all aspects of the work. All persons designated as authors qualify for authorship, and all those who qualify for authorship are listed.

### Sources of funding

The work was supported by funding from the British Heart Foundation (PG/12/23/29475 and PG/15/14/31311).
